# H_2_ controller design for a kestrel-inspired ornithopter operating in extreme weather

**DOI:** 10.1371/journal.pone.0342245

**Published:** 2026-02-12

**Authors:** Saddam Hussain Abbasi, Abdul Khader Jilani Saudagar, Nouman Abbasi

**Affiliations:** 1 Department of Electrical and Computer Engineering, SS CASE IT, Islamabad, Pakistan; 2 Information Systems Department, College of Computer and Information Sciences, Imam Mohammad Ibn Saud Islamic University (IMSIU), Riyadh, Saudi Arabia; 3 Department of Mechanical Engineering, National University of Sciences and Technology, Islamabad, Pakistan; King Fahd University of Petroleum & Minerals, SAUDI ARABIA

## Abstract

Unsteady atmospheric disturbances significantly compromise the flight stability of ornithopters, necessitating advanced turbulence-mitigation strategies. Drawing inspiration from the kestrel’s covert feathers, this study presents the modeling, control synthesis, and performance evaluation of a kestrel-inspired ornithopter equipped with an active covert-feather-based Gust Mitigation System (GMS). A reduced-order multibody bond-graph model (BGM) is derived from the full flapping-wing dynamics, capturing the coupled aero-elastic interaction between the main body, rigid wings, propulsion system, and feather actuation mechanism. Stability analysis reveals the presence of unstable internal dynamics, motivating the design of an H₂ optimal controller to ensure robust stability and fast disturbance rejection. The controller’s performance is evaluated against a Linear Quadratic Regulator (LQR) under vertical gust inputs ranging from 0 m/s to 20 m/s using MATLAB/Simulink simulations. Quantitative results indicate that the H₂-augmented GMS installed ornithopter reduces gust-induced forces by up to 32% and achieving faster state convergence within 1.1 seconds. The simulation results exhibit close agreement with previously reported findings, validating the fidelity of the proposed model and control framework. This work represents the first complete kestrel-inspired ornithopter integrating a bio-inspired GMS with H₂ optimal control, offering a validated and scalable foundation for next-generation adaptive ornithopters capable of maintaining stability in unsteady atmospheric environments.

## 1. Introduction

Small unmanned aerial vehicles (UAVs) face their greatest operational challenge when flying close to the earth’s surface, where the atmospheric boundary layer (ABL) produces strong turbulence and unpredictable gusts. These rapid fluctuations can destabilize flight by disturbing velocity, attitude, and control responses [1]. Addressing these vulnerabilities requires equipping UAVs and ornithopters with active gust mitigation strategies, supported by bio-inspired closed-loop control, to ensure stable and reliable flight in turbulent environments [2].

A wide range of traditional approaches for handling external disturbances in ornithopters have been explored in previous studies. The authors in [3] examined advances in reactive inertial sensors and reported significant time delays and slow response when using a single sensor for flight control in gusty conditions. Their findings highlight the necessity of employing multi-sensor systems for effective attitude control. In a subsequent study [4], the authors demonstrated that conventional reactive attitude sensors exhibit slow response times for attitude control in turbulent environments. To address these delays, they developed novel biologically inspired sensors capable of providing phase-advanced disturbance information, thereby enhancing actuator response time and improving stability during severe weathers.

A passive stabilization method using a cylindrically shaped aerodynamic device was presented in [5] to enhance the resilience of flapping-wing micro-aerial vehicles (FMAVs) without the need for sensors or feedback control. The device generated uniform corrective moments, enabling recovery from pitch and roll disturbances of up to ±40° and –75°, respectively, as well as vertical displacements of nearly 160 mm within one second. Compared with conventional cross-arm dampers, this approach extended passive hover duration from 1–2 seconds to over 15 seconds. Furthermore, tests under light gust conditions (up to 2.6 km/h) confirmed its effectiveness as a lightweight inspired disturbance rejection solution. Another investigation highlights the use of vortex generators to alleviate wing loads during high-speed flight, aiming to prevent wing-tip stall and reduce aerodynamic forces during turbulence and gust events [6].

Several other designs have been made to improve UAV avionics to attain higher performance in the turbulent airflows. Ratti et al. [7] presented the possibility of equipping UAVs with avionics that can achieve stabilization performance similar to large sized aircrafts. A Micro Architecture and Control (MARC) avionics design is developed with substantial improvements in sensory delays, weight reduction and power consumption to cater for the size constraints of UAVs. However, all these conventional gust alleviation strategies have primarily been evaluated for fixed-wing aircraft or traditional UAVs, and their applicability to ornithopters remains largely unexamined.

In the past ten years, biomimetics has advanced swiftly to tackle engineering challenges. Numerous biologically motivated designs have also been proposed to address turbulence [8]. Authors in [9] introduced bio-inspired flow sensors modeled after birds’ primary and secondary feathers for fixed-wing UAVs, demonstrating their effectiveness in mitigating gust effects. These artificial feathers were mounted on both the upper and lower wing surfaces, functioning simultaneously as sensors and actuators. The implemented design showed notable gust alleviation in the prototype fixed-wing UAV whereas its implementation for ornithopters remains an open area.

In addition to active gust alleviation design techniques several researches have also been done till date to stabilize UAVs using linear and non-linear control strategies [10–12]. Yu et al. [13] in their recent study address the problem of recovery flight in flapping-wing micro-aerial vehicles (FWMAVs) subjected to extreme attitudes caused by aggressive maneuvers and wind disturbances. The authors propose a reinforcement learning (RL)-based controller that enables the vehicle to regain stable flight while minimizing angular acceleration. To ensure sustained stability after recovery, a hybrid control strategy is introduced by combining the RL controller with a proportional-derivative (PD) controller. Simulation results demonstrate the effectiveness of both the RL and hybrid approaches in managing recovery and stabilization under challenging flight conditions, with future work aimed at real-world implementation.

Geder et al. [14] developed a six-degree-of-freedom (6-DOF) model of a flapping-wing UAV (FUAV) that incorporated the dynamics of the main body, wings, sensors, and shape-memory-alloy-based actuators. Sensory feedback control algorithms were designed and implemented in simulation, where the four control modules adjusted the wing stroke amplitude, stroke plane angle, and mean position. An extended Kalman filter was employed to enhance attitude estimation and stabilize the FUAV. Furthermore, PD and proportional integral derivative (PID) controllers were used to track desired responses in hovering, forward flight, and turning maneuvers. The simulated results demonstrated satisfactory performance across all flight modes.

A recent study [15] introduces a multi-level optimization model predictive control framework to balance computational load and control accuracy in FWMAVs. Using a quasi-steady aerodynamic model to estimate forces and moments, the method combines traditional model predictive controller (MPC) for path tracking with an added layer to fine-tune kinematics. The proposed controller outperforms proportional integral derivative (PID) and standard MPC by offering better tracking and quicker response under gusty conditions. Simulations show robust performance, though higher gusts still cause tracking deviations, highlighting the need for further stability research.

Coleman and Benedict [16] designed a bioinspired robotic hummingbird featuring a dual-wing configuration optimized for stable hovering. Pitch stabilization was achieved using a proportional derivative (PD) controller, with gain tuning focused on minimizing oscillations and ensuring prompt recovery. The system was evaluated under gust-like disturbances, showing fast stabilization and minimal positional drift. Although PID feedback was considered, it played a limited role in performance evaluation. The results highlight the effectiveness of lightweight, nature-inspired control for gust disturbance rejection.

Bluman et al. [17] demonstrated the effectiveness of sliding mode control (SMC) for a bumblebee model hovering in the pitch plane under severe uncertainties, including gusts, wing damage, and non-equilibrium initial conditions. The control inputs included flapping amplitude (vertical force), stroke plane angle (horizontal force), and flapping offset angle (pitch moment). In all scenarios, the controller achieved the desired response in under 1 s (within three flapping cycles). Key SMC features discussed included finite-time convergence, asymptotic state stability, and reduced control chattering via integrated switching. Although no method overwhelmingly dominated, classical SMC provided faster input computation, quicker gust rejection, and slightly smoother cycle-to-cycle control behavior.

Zheng et al. [18] introduced a tailless flapping-wing robot featuring three independently actuated wing pairs and bio-inspired elastic passive legs, enabling both aerial and ground mobility. A cascaded PID control loop with a Special Euclidean group in three dimensions (SE (3)) based geometric tracking controller was implemented for stable hovering and precise trajectory tracking. Although yaw control was underactuated, the system-maintained stability and maneuverability. While not specifically tested under strong gusts, the design demonstrated robustness to small disturbances.

Existing control strategies have proven effective primarily for handling mild disturbances and low-speed winds, but they struggle to manage stronger gusts effectively. This is largely because most approaches attempt gust mitigation in isolation, without incorporating active aerodynamic design elements to support control performance. Study in [19] also observed this limitation, noting that single-controller schemes are inadequate for flapping-wing drones operating in dynamic and highly nonlinear environments. They recommend integrating active structural features such as feather-like mechanisms, or employing a multi-layered control framework with parallel modules to achieve more robust gust rejection.

**Fig 1:**
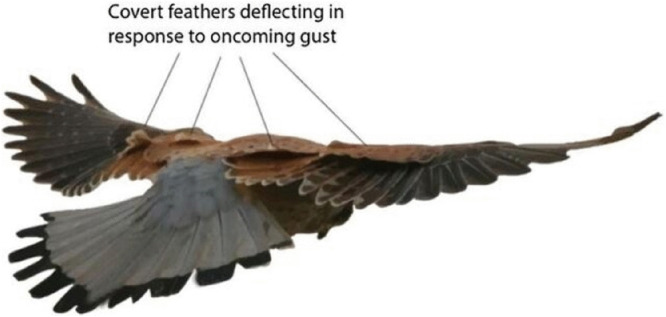
Kestrel’s covert feathers. Covert feathers deflect in response to vertical gusts.

This challenge has prompted a shift in focus toward natural flyers, whose evolved strategies may hold the key to effective gust handling. Extensive studies on kestrels have shown that they often switch to intermittent flight patterns when encountering gusty winds and turbulent atmospheric conditions. During these non-flapping phases, kestrels typically either glide or loiter in the air. It is in these moments that the covert feathers become active, deploying automatically to mitigate the adverse effects of turbulence and gusts. This behavior is illustrated in Fig 1 [20].

Inspired by avian covert feathers, authors in [21] proposed a biomimetic gust alleviation system (GAS) for the wing of a flapping-wing UAV. This system incorporates feathers seamlessly integrated into the UAV’s wing structure. The GMS is selectively activated under turbulent conditions to dissipate incoming gusts, while remaining fixed during calm weather to preserve the wing’s aerodynamic profile. Later work [22] presents a robust controller design for a wing equipped with GAS, successfully stabilizing its dynamics. These approaches provide several flight benefits, notably improved stability in adverse weather environments. However, these works are confined to a single wing equipped with GAS and their applicability to a complete UAV needs to be studied.

Authors in [23,24] showed that the feather-equipped ornithopter is dynamically unstable and necessitates an active controller design to achieve stable and robust flight during turbulence and gusts. In this study, we build upon these findings and present a novel design of $H_{\text{2}}$ controller for a complete ornithopter integrated with a kestrel-inspired covert feather-based GMS on both wings.

In the context of this study, gusty conditions refer to vertical atmospheric disturbances that disrupt the periodic aerodynamic loading on the flapping wings. For lightweight flapping-wing UAVs such as the Festo Flapping Bird, wind fluctuations exceeding 3 m/s are generally considered gusts that can significantly affect flight stability. Based on available experimental data and prior literature, vertical gust amplitudes up to 20 m/s represent strong yet physically realistic outdoor conditions for ornithopters of comparable scale [25]. Accordingly, this vertical gust speed range has been adopted in the present simulations to assess the controller’s robustness under severe but practical disturbances. Evaluation under more lateral and longitudinal extreme gusts (> 20 m/s) is reserved for future extensions of this research.

The $H_{\text{2}}$ control approach is adopted in this work due to its capability to minimize the total energy of gust-induced disturbances transmitted to the ornithopter states and outputs. Compared with Model Predictive Control (MPC), which requires iterative optimization at every time step, and $H_{\infty }$ control, which tends to yield conservative performance by prioritizing worst-case robustness, the $H_{\text{2}}$ framework provides a balanced trade-off between computational efficiency and disturbance-attenuation accuracy. In contrast to reinforcement-learning approaches that demand large datasets and heavy onboard computation, the $H_{\text{2}}$ formulation ensures predictable stability and lower implementation cost. Overall, these properties make $H_{\text{2}}$ especially suitable for real-time implementation on low-power ornithopters, where rapid adaptation to gusts is essential for stable flight. Finally, the key contributions of this work include:

- We develop a reduced-order longitudinal bond-graph model of a complete flapping-wing ornithopter equipped with a kestrel-inspired covert-feather based Gust Mitigation System (GMS) on both wings, capturing the coupled aero-elastic interactions needed for control synthesis.
- We formulate and implement an $H_{\text{2}}$ controller specifically tailored for vertical gusts up to 20 m/s, minimizing disturbance-to-output energy and ensuring robust stabilization of the inherently unstable internal dynamics; performance is benchmarked against an LQR baseline under identical disturbance scenarios.
- We provide quantitative validation of the $H_{\text{2}}$-augmented GMS, including step and sinusoidal-gust evaluations, demonstrating up to 32% reduction in gust-induced effects and fast, well-damped convergence, thereby establishing a validated, bio-inspired control framework for resilient ornithopter flight in unsteady atmospheres.

Collectively, this represents, to the best of our knowledge, the first complete kestrel-inspired ornithopter that integrates an active covert-feather GMS with $H_{\text{2}}$ control, bridging bio-inspired aerodynamics and modern optimal control for practical gust mitigation.

The remainder of the paper is organized as follows: Section 2 describes the architecture of the complete ornithopter equipped with the GMS and details the formulation of its reduced-order longitudinal bond graph model. Section 3 presents control oriented reduced order model of the GMS installed ornithopter while the stability analysis is covered in section 4. Section 5 outlines the design of the $H_{\text{2}}$ controller for gust mitigating ornithopter. Section 6 discusses the simulation results, and section 7 concludes the paper.

## 2. System architecture and modeling

**Fig 2:**
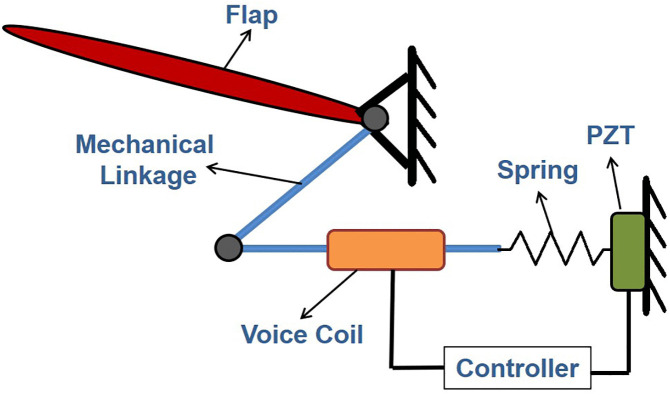
Electromechanical covert feather [22]. During gusts flap transfers motion to PZT through mechanical linkage and spring. PZT generates voltage and feds it to controller for further generation of control input which is fed through voice coil to flap which deflects to transpire gust.

The Festo Flapping Bird [26] is selected as the baseline ornithopter for this study. The proposed ornithopter is composed of subsystems namely the main body, motors, the flapping mechanism, rigid wings and GMS. The GMS incorporates a total of 8 electromechanical (EM) covert feathers and is installed in each ornithopter wing. Each EM covert feather includes a flap, mechanical linkage, voice coil actuator, bio-inspired controller, spring, and a piezoelectric transducer (PZT). The configuration of the EM feathers is illustrated in Fig 2. The flap rotates under a vertical gust and transfers motion through the spring and mechanical link to the PZT. The PZT generates a voltage proportional to the gust and sends it to the bio-mimetic controller, which computes the control input. This current drive the voice coil actuator, whose shaft applies force back on the flap via the linkage to create the required deflection. Thus, the gust transpires with minimal interaction on the wing.

Bond graphs offer a powerful, domain-independent graphical approach for modeling the dynamic behavior and energy exchange in physical systems. In contrast to conventional methods that are typically confined to a single domain, bond graphs provide a unified framework capable of representing systems across mechanical, electrical, hydraulic, thermal, and other energy domains. This modeling technique is particularly well-suited for complex, multi-domain systems like ornithopters, where coordinated behavior among mechanical structures, actuators, and aerodynamic forces must be accurately captured. Moreover, bond graphs naturally lend themselves to generating state-space representations, facilitating seamless integration with control design and simulation processes [27].

Given the multi-domain nature of the proposed ornithopter, bond graph modelling is employed in this study to derive its mathematical representation. 20-SIM simulation software is used for development of BGM. The ornithopter is composed of subsystems namely the main body, propulsion system (motors and flapping mechanism), rigid wings and GMS.

### 2.1. Rigid body

The ornithopter’s body is presented as 6-DOF rigid structure which can complete both translational & rotational motions. The investigation of rigid body’s motion generates equations appended below derived from the Euler’s equations [27]. The state matrix contains generalized momentum p_x_, p_y_, p_z_, p_jx_, p_jy_, and p_jz_ at every inertia element.


$$\dot{{\text{p}}_{\text{x}}}={\text{F}}_{\text{x}}+{m\omega }_{\text{z}}\frac{{\text{P}}_{\text{y}}}{m}-{m\omega }_{\text{y}}\frac{{\text{P}}_{\text{z}}}{m}$$ (1)

$$\dot{{\text{p}}_{\text{y}}}={\text{F}}_{\text{y}}+{m\omega }_{\text{x}}\frac{{\text{P}}_{\text{z}}}{m}-{m\omega }_{\text{z}}\frac{{\text{P}}_{\text{x}}}{m}$$ (2)

$$\dot{{\text{p}}_{\text{z}}}={\text{F}}_{\text{z}}+{m\omega }_{\text{y}}\frac{{\text{P}}_{\text{x}}}{m}-{m\omega }_{\text{x}}\frac{{\text{P}}_{\text{y}}}{m}$$ (3)

$$\text{p}\dot{{\text{J}}_{\text{x}}}=\tau_{\text{x}}+{\text{J}}_{\text{y}}\omega_{\text{y}}\frac{{\text{PJ}}_{\text{z}}}{{\text{J}}_{\text{z}}}-{{\text{J}}_{\text{z}}\omega }_{\text{z}}\frac{{\text{PJ}}_{\text{y}}}{{\text{J}}_{\text{y}}}$$ (4)

$$\text{p}\dot{{\text{J}}_{\text{y}}}=\tau_{\text{y}}+{\text{J}}_{\text{z}}\omega_{\text{z}}\frac{{\text{PJ}}_{\text{x}}}{{\text{J}}_{\text{x}}}-{{\text{J}}_{\text{x}}\omega }_{\text{x}}\frac{{\text{PJ}}_{\text{z}}}{{\text{J}}_{\text{z}}}$$ (5)

$$\text{p}\dot{{\text{J}}_{\text{z}}}=\tau_{\text{z}}+{\text{J}}_{\text{x}}\omega_{\text{x}}\frac{{\text{PJ}}_{\text{y}}}{{\text{J}}_{\text{y}}}-{{\text{J}}_{\text{y}}\omega }_{\text{y}}\frac{{\text{PJ}}_{\text{x}}}{{\text{J}}_{\text{x}}}$$ (6)

where $\dot{p_{x}},\dot{p_{y}},\dot{p_{z}}$ are the derivatives of linear momenta, $\dot{{pj}_{x}},\dot{{pj}_{y}},\dot{{pj}_{z}}$ are the derivatives of angular momenta, F_x_, Fy, Fz are forces acting along the body-axes, τ_x_, τ_y_, τ_z_ are torques about the body-axes, m, $J_{x},J_{y},J_{z}$ are linear and rotational inertias and $\omega_{x},\omega_{y},\omega_{z}$ are angular-velocity components about the body-axes.

### 2.2. Propulsion system

The propulsion system comprises batteries, motors and a slider-crank mechanism. The two DC motors are driven by batteries and are comprised of an armature having resistance and inductance and an electro-mechanical coupling. The ornithopter’s flapping movement is achieved by slider-crank mechanism actuated by DC motors. The reciprocating motion is received and transmitted through a crank shaft which helps achieve transformation of rotational motion in to a reciprocating motion and also vice-versa [28].

### 2.3. GMS equipped wings

Wings’ dynamics are demonstrated as a rigid beam in transverse motion having a pivoted end. The wing’s perpendicular displacement at the end point is obtained by:


$$\begin{array}{c} y=lsin\theta \end{array}$$ (7)

where *l* is wing span, *y* is displacement and *θ* is flapping angle. The effort-flow relationship can be given as [29]:


$$\begin{array}{c} V_{y}=(lcos\theta )\omega \end{array}$$ (8)

$$\begin{array}{c} \left(x_{\text{1}}\text{cos}\theta \right)F=\tau \end{array}$$ (9)

where *τ* is torque, *F* is force and *V*_*y*_ is vertical velocity.

**Fig 3:**
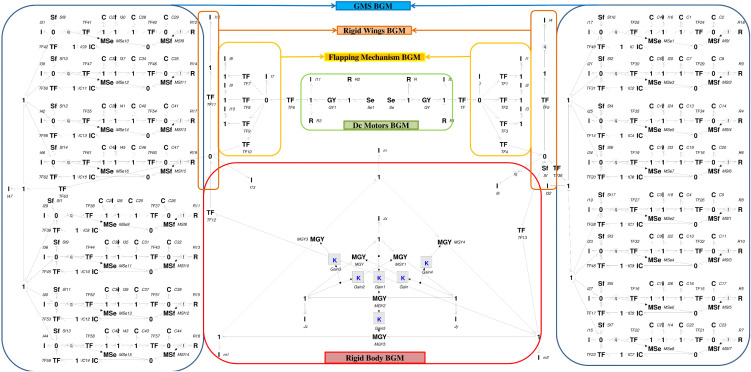
Bond graph model of a complete ornithopter. Bond graph model of the proposed ornithopter is composed of bond graph models of subsystems namely the main body, motors, the flapping mechanism, rigid wings and GMS.


The wings of ornithopter are equipped with kestrel-inspired feather-based GMS. Model of a single EM covert feather is developed using the component diagram of EM feather given in Fig 1 and this is further utilized to form the BGM of a GMS comprising 8 EM feathers.The mathematical equations for a single EM feather, derived from the BGM depicted in Fig 3, are presented in Equations (10) to (17).

$$\dot{p_{\text{1}}}=ic\cdot p_{\text{3}}+ic\cdot q_{\text{3}}$$ (10)

$$\dot{q_{\text{1}}}=\frac{\text{1}}{I_{\text{1}}}\cdot p_{\text{2}}$$ (11)

$$\dot{p_{\text{2}}}=\frac{ic}{l}p_{\text{3}}+\frac{ic}{l}q_{\text{3}}-\frac{\text{1}}{C}q_{\text{1}}-\frac{\text{1}}{C_{\text{1}}}q_{\text{2}}-\frac{m}{C_{\text{2}}}q_{\text{4}}$$ (12)

$$\dot{q_{\text{2}}}=\frac{\text{1}}{I_{\text{1}}}\cdot p_{\text{2}}$$ (13)

$$\dot{p_{\text{3}}}=q_{\text{5}}$$ (14)

$$\dot{q_{\text{3}}}=S_{f}-\frac{\text{1}}{l\cdot I_{\text{1}}}p_{\text{2}}-\frac{\text{1}}{I}p_{\text{1}}$$ (15)

$$\dot{q_{\text{4}}}=\frac{m}{I_{\text{1}}}p_{\text{2}}-\frac{R}{C_{\text{2}}}q_{\text{4}}$$ (16)

$$\dot{q_{\text{5}}}=\frac{\text{1}}{l\cdot I_{\text{1}}}p_{\text{2}}$$ (17)

The state variables in Equations (10) to (17) consist of p1, p2, p3 which represent the generalized momentum associated with the inertial elements, and q1, q2, q3, q4 which denote the generalized displacements corresponding to the compliance elements. I is mass of feather flap, Sf is gust velocity on flap, IC is voice coil actuator compliance and stiffness, R is resistance between amplifier and PZT, I1 is mass of stack, C1 is PZT spring stiffness, C2 is PZT equivalent capacitance, TF1 is electromechanical coupling ratio, C is stiffness of spring, TF is transformer ratio of mechanical linkage.

### 2.4. Multibody model of a complete ornithopter

The model of complete ornithopter is established by connecting the BGM of sub-systems via appropriate junctions and is depicted in Fig 3. For detailed component level development of a complete ornithopter model further reading of author’s previous work is suggested [23,24]. Parameters of the complete bond graph model are provided in the Supporting Information (S1–S6 Tables). The state vector $\overset{⇀}{x}(t)$ comprises the momenta of the I-elements and the generalized displacements of the C-elements. Linearization about the hover trim condition yields a 132-state model which captures the complete 6-DOF rigid-body dynamics of the ornithopter installed with GMS in both wings. Since the focus of this study is on flight control synthesis, the analysis is restricted to the longitudinal plane. In this way, only the forward velocity, vertical velocity, pitch angle, and pitch rate are retained, while the lateral directional states are excluded. The resulting subsystem provides a direct pathway to the conventional four-state longitudinal model, which is presented in the next section.

The model is trimmed about a hover equilibrium condition, representing the most dynamically sensitive regime of the ornithopter. Extensive studies on kestrels show that these birds often shift to intermittent flight; gliding or loitering, when encountering gusty or turbulent air, relying on covert-feather modulation to maintain stability as explained in Fig 1. Hover therefore provides a biologically realistic and conservative basis for control synthesis: if stability can be achieved under this condition, it can be extended to near-forward flight with minor gain adaptation. The linearization was performed about the following nominal hover parameters: forward velocity of 0 m/s, vertical velocity of 0 m/s, pitch angle of 2.5°, and pitch-rate of 0 rad/s. The covert feather displacements at trim were taken as 0 m, with zero net aerodynamic load on each feather module and zero control input. These values correspond to the static equilibrium achieved by the bond-graph model under no-gust conditions.

In this research, the scope is limited to vertical gust excitation, which primarily influences the lift dynamics of the ornithopter. The comprehensive modeling of a GMS-equipped ornithopter that incorporates all aerodynamic and structural interactions is highly complex; therefore, several simplifying assumptions are introduced to maintain model tractability. Following the approach of recent studies [30,31], a range of secondary aerodynamic effects, such as wing-wake interaction, rotational inertia, circular rotation, rotational lift, leading-edge vortex generation, thrust, drag, viscous friction, and added mass are neglected. These effects have relatively minor influence within the moderate Reynolds number regime considered here and can be incorporated in future, higher-fidelity models. The present reduced-order bond-graph model thus captures the dominant dynamics responsible for gust structure coupling and provides a practical foundation for control-law design and performance evaluation.

## 3. Control oriented model

For the purpose of controller design, the reduced four-state longitudinal model of the GMS-equipped ornithopter is employed. This representation, obtained at the hover trim condition, captures the dominant forward and vertical velocity dynamics together with the pitch motion, making it well suited for stability analysis and control synthesis. The model is formulated in a linear time-invariant (LTI) state-space form and serves as the basis for the subsequent $H_{\text{2}}$ controller design. The state vector x, control vector u and the output vector y are as follows:

*x* = [*u w* θ *q*]^*T*^, *u* = [*φ*_*o*_ *α*_*m*_ *α*_*o*_]^*T*^, *y* = [*u w* θ]^*T*^. The detail of these vectors is given in Table 1. Here, the flapping stroke offset $\varphi_{o}$ biases thrust and primarily affects the forward dynamics, the pitch angle magnitude $\alpha_{m}$ governs lift and hence the vertical dynamics, and the pitch angle offset $\alpha_{o}$ influences the pitching moment of the GMS-equipped ornithopter. The resulting LTI system can be represented in state space form as: -

**Table 1:** Parameters of the LTI Model.

Parameters	Description	Units	Parameters	Description	Units
u	Body forward velocity	m/s	$\varphi_{o}$	Flapping stroke offset	rad
w	Body vertical velocity	m/s	$\alpha_{m}$	Pitch angle magnitude	rad
θ	Body pitch angle	rad	$\alpha_{o}$	Pitch angle offset	rad
q	Body pitch rate	rad/s			


$$\begin{array}{c} \dot{\overset{⇀}{x}}(t)=A\cdot \overset{⇀}{x}(t)+B\cdot \overset{⇀}{u}(t)\\ \overset{⇀}{y}(t)=C\cdot \overset{⇀}{x}(t)+D\cdot \overset{⇀}{u}(t) \end{array}$$ (18)

$$A=[\begin{array}{cccc} -\text{5}.\text{9}\text{8}\text{9} & \text{0} & \text{0} & -\text{0}.\text{0}\text{6}\text{9}\\ \text{0} & -\text{1}.\text{9}\text{8} & -\text{8}.\text{9}\text{1} & \text{0}.\text{0}\text{0}\text{4}\\ \text{0} & \text{0} & \text{0} & \text{1}.\text{3}\text{0}\\ \text{0} & \text{1}\text{4}\text{9}.\text{3}\text{4} & \text{0} & -\text{0}.\text{2}\text{9}\text{8} \end{array}]$$

$$B=[\begin{array}{ccc} \text{0} & \text{1}\text{7}.\text{0} & \text{0}\\ \text{0} & \text{0} & -\text{1}\text{2}.\text{7}\\ \text{0} & \text{0} & \text{0}\\ \text{1}\text{4}\text{8}\text{3} & \text{0} & \text{5}\text{9}\text{7} \end{array}]$$

$$\text{C}=[\begin{array}{cccc} \text{1} & \text{0} & \text{0} & \text{0}\\ \text{0} & \text{1} & \text{0} & \text{0}\\ \text{0} & \text{0} & \text{1} & \text{0} \end{array}]\text{D}=\text{zeros}(\text{3},\text{3})$$

## 4. System stability study

**Fig 4:**
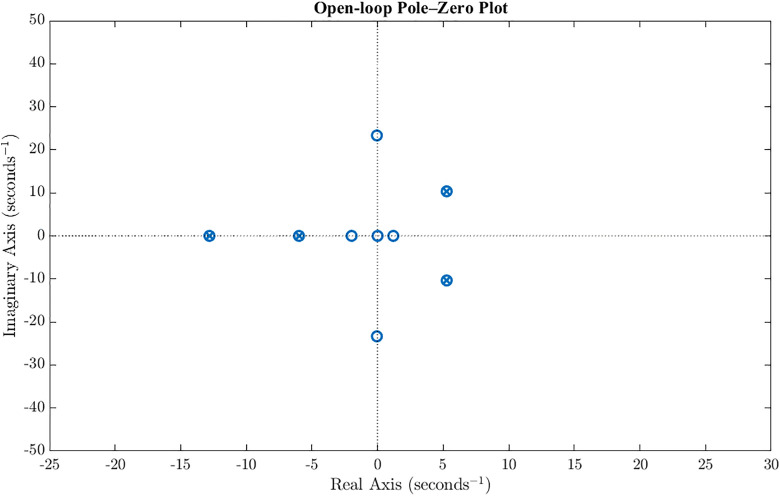
Open loop pole zero plot. Open loop pole zero plot of the ornithopter shows that multiple poles are in right half plan and therefore depicts unstable internal dynamics of the ornithopter.

**Fig 5:**
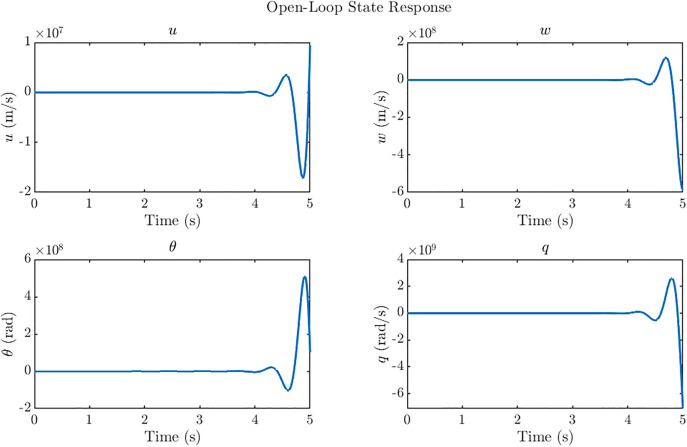
Ornithopters’ open loop states response. Open loop states response of the ornithopter is diverging and therefore depicts unstable internal dynamics of the ornithopter.

**Fig 6:**
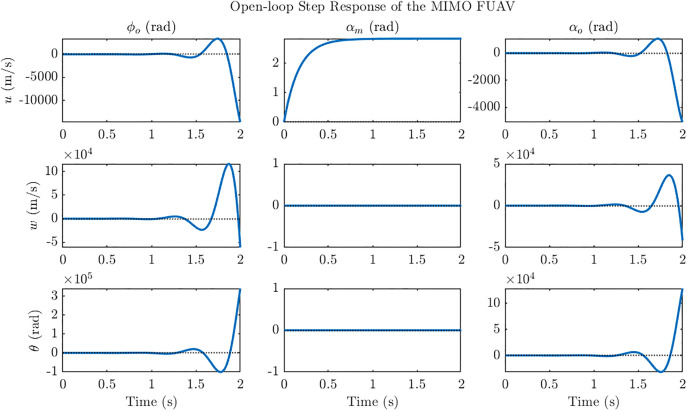
Open loop step response of the multi input multi output (MIMO) system. Open loop step response of the MIMO ornithopter system is diverging and therefore depicts unstable internal dynamics of the ornithopter.

Stability analysis of the reduced-order system is presented in this section. The poles of the open loop system are located at: −5.98, −12.81, 5.26 ± 10.35j. The presence of a conjugate pair of poles in right-half-plane as shown in Fig 4 indicates that the system is inherently unstable. Fig 5 illustrates the unstable state response of the open-loop system which shows diverging state response and instability in the system. Moreover, the open-loop step response of the multi-input multi-output (MIMO) ornithopter, shown in Fig 6, again highlights the strong instability and severe cross-coupling between states, with large unbounded excursions in response to step commands, thereby confirming the need for designing a stabilizing controller to ensure attitude stability under gust disturbances. In the open-loop dynamics, instability primarily originates from the pitch subsystem (θ, *q)* as the coupling between the angular displacement and pitch rate introduces positive feedback in the moment equation. The heave state *w* remains marginally stable but amplifies oscillations when coupled with the pitching motion. In contrast, the forward velocity *u* exhibits fast, well-damped behavior and is not a major source of instability.

## 5. *H*_*2*_ controller design

**Fig 7:**
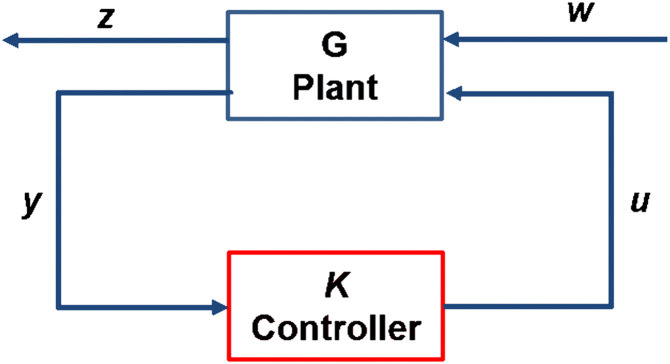
*H*_*2*_ control block diagram. H₂ control block diagram showing ornithopter plant *G* regulated by controller *K* in feedback loop.

In this section, we develop the GMS-equipped ornithopter controller based on optimal control principles, specifically employing the *H*_2_ control approach. The closed-loop *H*_2_ configuration is shown in Fig 7. The *H*_2_ control framework is an optimal design methodology that improves system performance by minimizing the overall energy of the closed-loop response to disturbances and noise [32]. By minimizing the *H*_2_ norm of the closed-loop transfer function, the controller effectively reduces the energy of the system response to gust disturbances, thereby enhancing stability and efficiency in turbulent airflows. We choose *H*_2_ control in this study because it is particularly suitable for gust mitigation as it directly minimizes the influence of energy-like gust inputs on the UAV dynamics, ensuring efficient attenuation of gust-induced oscillations without excessive control effort.

In Fig 7, *G* represents the plant, *K* denotes the controller, *y* is the measurement signal, *w* represents the disturbance, *u* is the control input, and *z* is the vector comprising all regulated signals. The *H*_2_ controller is designed to minimize the *H*_2_ norm [33]. The design process begins by formulating the transfer function, expressed as follows: -


$$G(s)=[\begin{array}{ccc} A & B_{w} & B_{u}\\ C_{y} & \text{0} & D_{yu}\\ C_{m} & D_{mw} & o \end{array}]$$ (19)

There are certain assumptions that are required to be met before further development of the *H*_2_ controller [34]. These assumptions are appended below: -

- (*A,B*_*u*_) is stabilizable and (*C*_*m*_*,A*) is detectable.
-
${D_{yu}}^{*}\times [\begin{array}{cc} C_{y} & D_{yu} \end{array}]=[\begin{array}{cc} \text{0} & I \end{array}]$

-
$\left[\begin{array}{c} B_{w}\\ D_{mw} \end{array}]\times {D_{mw}}^{*}=[\begin{array}{c} \text{0}\\ I \end{array}\right]$

- $\left[\begin{array}{cc} A-j\omega I & B_{u}\\ C_{y} & D_{yu} \end{array}\right]$ has full column rank for all ω
- $\left[\begin{array}{cc} A-j\omega I & B_{w}\\ C_{m} & D_{mw} \end{array}\right]$ has full column rank for all ω

Meeting the above assumptions will guarantee the development of *H*_2_ controller properly. The general solution of *H*_2_ controller comprises two Hamiltonian matrices that are given in equation (20) and (21) [35].


$$H_{\text{2}}=[\begin{array}{cc} A-B_{u}{\left({D_{yu}}^{*}\times D_{yu}\right)}^{-\text{1}}{D_{yu}}^{*}C_{y} & -B_{u}{\left({D_{yu}}^{*}\times D_{yu}\right)}^{-\text{1}}{B_{u}}^{*}\\ -{C_{y}}^{*}\{I-D_{yu}{\left({D_{yu}}^{*}\times D_{yu}\right)}^{-\text{1}}{D_{yu}}^{*}\}C_{y} & {-(A-B_{u}{\left({D_{yu}}^{*}\times D_{yu}\right)}^{-\text{1}}{D_{yu}}^{*}C_{y})}^{*} \end{array}]$$ (20)

$$J_{\text{2}}=[\begin{array}{cc} (A-B_{w}{D_{mw}}^{*}{\left({D_{mw}\times D_{mw}}^{*}\right)}^{-\text{1}}{C_{m})}^{*} & -{C_{m}}^{*}{\left({D_{mw}\times D_{mw}}^{*}\right)}^{-\text{1}}C_{m}\\ -B_{w}\{I-{D_{mw}}^{*}{\left({D_{mw}\times D_{mw}}^{*}\right)}^{-\text{1}}D_{mw}\}{B_{w}}^{*} & A-B_{w}{{D_{mw}}^{*}\left({D_{mw}\times D_{mw}}^{*}\right)}^{-\text{1}}C_{m} \end{array}]$$ (21)

Once the above two Hamiltonian matrices are successfully calculated, then we need to compute the values of $X_{\text{2}}$ and $Y_{\text{2}}$ utilizing the equations (22) and (23).


$${X_{\text{2}}=Ric(H}_{\text{2}})$$ (22)

$${Y_{\text{2}}=Ric(J}_{\text{2}})$$ (23)

Finally, the $H_{\text{2}}$ controller gain is calculated as [35]: -


$${K_{\text{2}}=-\left({D_{yu}}^{*}\times D_{yu}\right)}^{-\text{1}}\times {(B_{u}}^{*}X_{\text{2}}+{D_{yu}}^{*}C_{y})$$ (24)

In summary, unlike LQR which minimizes a deterministic quadratic cost on state and control effort, and H∞ control, which minimizes the worst-case disturbance gain, the H₂ controller minimizes the average energy of the disturbance-to-error transfer function, resulting in smoother transient behavior under atmospheric gusts.

## 6. Results and discussions

**Fig 8:**
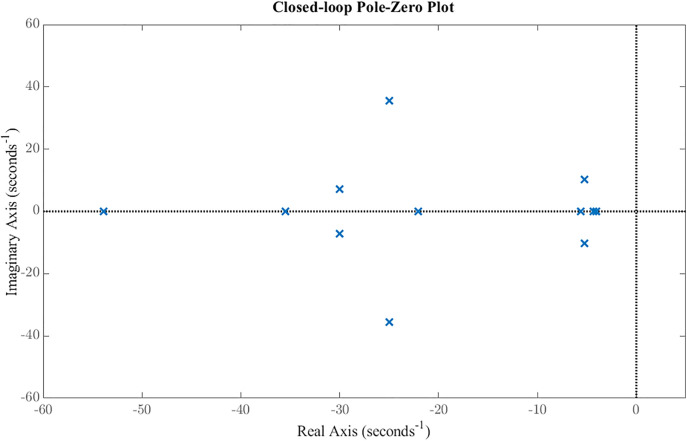
Closed loop pole-zero plot. All poles are present in left half plan and therefore depict that the *H*_*2*_ controller has successfully stabilized the unstable ornithopter.

The developed GMS-installed ornithopter model and its controller are simulated and analyzed in this section. An $H_{\text{2}}$ controller is synthesized to minimize the closed-loop energy from disturbance inputs to performance outputs, thereby achieving optimal tracking and disturbance rejection. $H_{\text{2}}$ controller’s design parameters used in this study are provided in the Supporting Information (S7 Table). The resulting controller is of order 13; after applying minimal realization using mineral in MATLAB, two high-frequency pole–zero cancellations are eliminated, yielding an effective 11^th^ order controller. Combined with the 4^th^ order GMS-installed ornithopter plant, the overall closed-loop system is of order 15. The closed-loop poles of the system are listed in Table 2, with the dominant poles located at approximately −5.26 ± 10.35i, governing the primary oscillatory dynamics of the ornithopter. Additional real poles near −4.07, −4.35, and −5.62 contribute to stable damping, while faster poles at −22.04 and −24.98 ± 35.63i ensure robustness and rapid decay of higher-frequency modes. Overall, all poles are present in left half plan and therefore depict that the $H_{\text{2}}$ controller has successfully stabilized the unstable ornithopter as shown in Fig 8.

**Table 2:** Closed-loop eigenvalues.

Poles	$\omega_{𝐧}$	ζ	Poles	$\omega_{𝐧}$	ζ
−24.98 ± 35.63i	43.511	0.574	−4.3583	4.358	1
−22.0428	22.042	1	−5.6228	5.622	1
−5.2679 ± 10.3559i	11.619	0.453	−4.0721	4.072	1

All simulations were conducted in MATLAB R2023a using the Control System and Robust Control Toolboxes. The continuous-time state-space models were numerically integrated with a fixed time step of 1 ms (Δt = 0.001 s) over a 5 s simulation horizon. The *lsim* solver (zero-order hold discretization of input signals) was employed for both LQR and H₂ closed-loop responses to ensure consistent time resolution and accurate transient capture. Figure generation and quantitative metrics (rise time, settling time, and overshoot) were computed directly from the simulated trajectories using custom functions.

**Fig 9:**
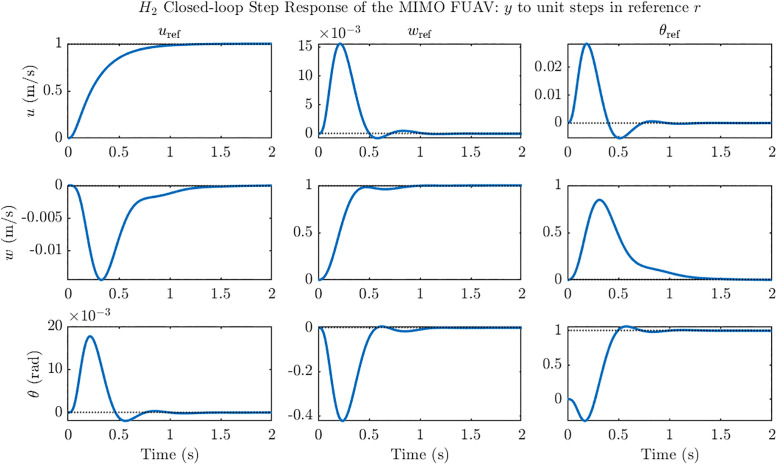
*H*_*2*_ Closed loop step response of the MIMO ornithopter system. MIMO step responses under H₂ control show stable tracking of u, w, and θ with settling times <1.5 s and minimal cross-coupling.

To further evaluate the multivariable performance of the designed $H_{\text{2}}$ controller, unit step commands were applied individually to each of the three reference channels, and the corresponding closed-loop outputs were recorded. The resulting multi-input multi-output (MIMO) step response, shown in Fig 9, highlights both the direct tracking behavior along the diagonal channels and the cross-coupling effects in the off-diagonal responses. All three commanded states: forward velocity u, vertical velocity w, and pitch angle θ exhibit stable convergence to their respective references with settling times under 1.5 s, while the cross-coupled outputs remain small and well-damped. This confirms that the $H_{\text{2}}$ controller not only stabilizes the unstable ornithopter dynamics but also ensures effective decoupling between the longitudinal states, thereby validating its suitability for multivariable trajectory tracking.

The closed-loop tracking performance of the ornithopter’s $H_{\text{2}}$ controller subjected to 20 m/s step gust is evaluated for three reference command scenarios:

- u *only step:* a forward velocity step of 0.20 m/s, $w_{ref}=\text{0}$ and $\theta_{ref}=\text{0}.$
- w *only step:* a vertical velocity step of 0.25 m/s, $u_{ref}=\text{0}$ and $\theta_{ref}=\text{0}.$
- *Simultaneous* u−w *step:* a combined forward velocity step of 0.20 m/s, a vertical velocity step of 0.25 m/s and $\theta_{ref}=\text{0}.$

**Fig 10:**
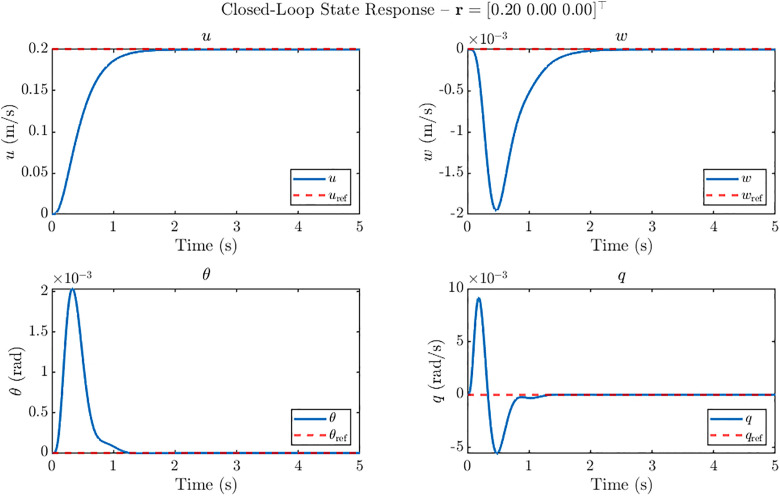
Closed loop state response of ornithopter at 20 m/s gust for reference tracking with r = [0.20 0 0]^T^. Step response for forward velocity command (u-only) under H₂ control showing accurate tracking with settling times <1.3 s, and negligible impact on w, θ, and q.

**Fig 11:**
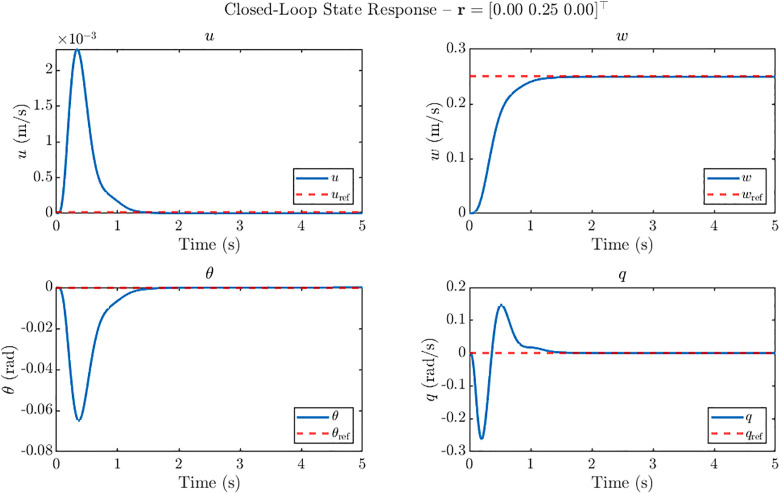
Closed loop state response of ornithopter at 20 m/s gust for reference tracking with r = [0 0.25 0]^T^. Step response for vertical velocity command (w-only) under H₂ control showing smooth tracking with settling times <1.4 s, negligible cross-coupling and well-damped θ and q oscillations.

**Fig 12:**
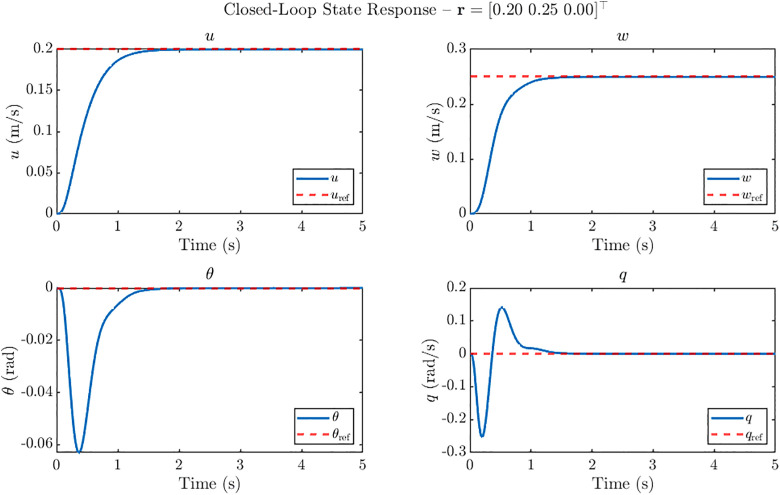
Closed loop state response of ornithopter at 20 m/s gust for reference tracking with r = [0.20 0.25 0]^T^. Simultaneous step responses for u and w under H₂ control showing accurate multi-input tracking with settling<1.4 s, negligible overshoot and well-damped θ and q dynamics.

For each case, the system states (uwθq), and the control inputs $\left(\varphi_{o}\alpha_{m}\alpha_{o}\right)$ are recorded over a 5 s simulation horizon. The resulting state trajectories for these reference inputs are shown in Figs 10–12, demonstrating the controller’s ability to achieve accurate tracking with minimal overshoot while maintaining stability.

Fig 10 shows that for the *u-only* step reference r = [0.20 0 0]^T^ the $H_{\text{2}}$ controller achieves smooth and accurate tracking with negligible overshoot and a settling time of approximately 1.3 s. The forward velocity *u* converges rapidly to the commanded value, while the vertical velocity *w* remains close to zero, exhibiting only a small transient dip during the initial acceleration. The pitch angle *θ* and pitch rate *q* show well-damped oscillations of the order of 10^−3^ rad and 10^−3^ rad/s, respectively, which vanish within 1.1 s. These results confirm the controller’s ability to precisely track forward-speed commands while preserving stability in the remaining states.

Fig 11 shows that for the *w-only* step reference r = [0 0.25 0]^T^, the $H_{\text{2}}$ controller successfully tracks the vertical velocity command with negligible overshoot and a settling time of approximately less than 1.4 s across the channels. The commanded *w* response rises smoothly to the target value, while the forward velocity *u* stays close to zero, exhibiting only a transient fluctuation of the order of 10^−3^ m/s. The pitch angle *θ* and pitch rate *q* display small, well-damped oscillations with peak magnitudes of about −0.07 rad and −0.27 rad/s, respectively, which decay rapidly without destabilizing the system. These results demonstrate that the controller maintains decoupled and stable performance during vertical velocity tracking.

For the simultaneous step commands r = [0.20 0.25 0]^⊤^, shown in Fig 12, the $H_{\text{2}}$ controller achieves accurate multi-input tracking with negligible overshoot and settling times of less than 1.4 s. Both the forward velocity *u* and vertical velocity *w* follow their respective references closely, without noticeable steady-state error. The pitch angle *θ* and pitch rate *q* exhibit modest oscillations, with peak amplitudes of about −0.06 rad and 0.26 rad/s, which decay quickly. These results confirm that the $H_{\text{2}}$ controller is effective in simultaneously regulating forward and vertical velocity commands, while maintaining stable and well-damped longitudinal dynamics of the GMS-equipped ornithopter.

**Fig 13:**
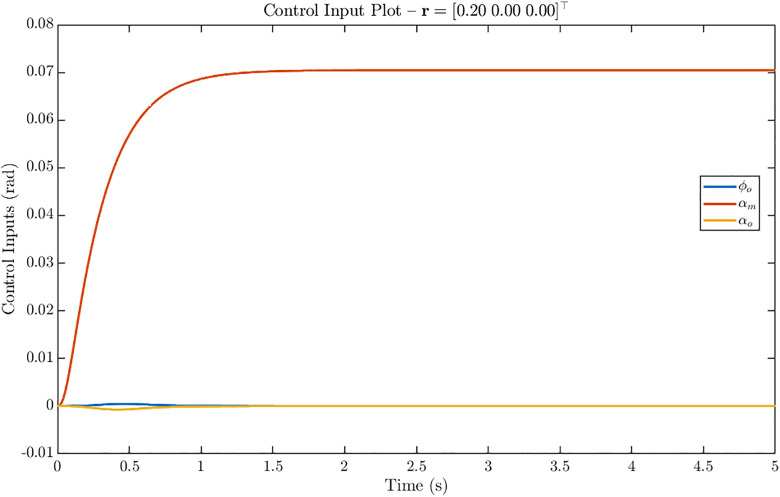
Control input response of ornithopter for reference tracking with r = [0.20 0 0]^T^. Control inputs for u-only step showing dominant αₘ actuation (≈ 0.07 rad) with minimal φₒ and αₒ activity, confirming efficient control allocation.

The control input trajectories corresponding to the *u-only* step reference r = [0.20 0 0]^T^ are shown in Fig 13. The dominant actuation is provided by the middle channel $\alpha_{m}$, which rises smoothly to a steady value of about 0.07 rad within the first second and remains constant thereafter. The outer channels $\varphi_{o}$ and $\alpha_{o}$ contribute minimally, exhibiting only small transient activity near the start before settling close to zero. The smooth profiles and absence of overshoot across all three channels confirm efficient control allocation with negligible coupling effects when tracking the forward velocity command.

**Fig 14:**
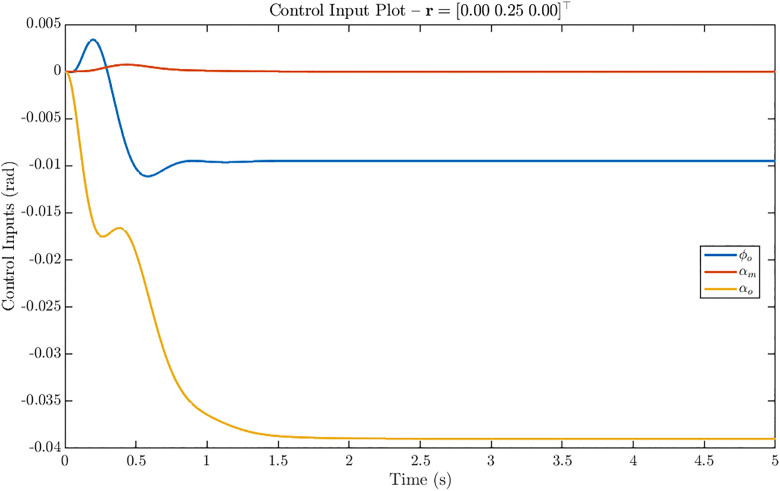
Control input response of ornithopter for reference tracking with r = [0 0.25 0]^T^. Control inputs for w-only step showing dominant αₒ (≈ −0.038 rad) with smaller φₒ contribution and negligible αₘ, indicating adaptive control allocation.

Fig 14 presents the control input profiles for the *w-only* step reference r = [0 0.25 0]^T^. In this case, the steady-state actuation is primarily provided by $\alpha_{o}$, which settles at approximately −0.038 rad, supported by a smaller contribution from $\varphi_{o}$ at about −0.01 rad. The $\alpha_{m}$ input remains near zero in steady state, showing only a small transient peak of 0.002 rad during the initial maneuver. Compared with the *u-only* case, the relatively higher activity in $\varphi_{o}$ and $\alpha_{o}$ highlights how the controller reallocates actuation authority depending on the commanded channel.

**Fig 15:**
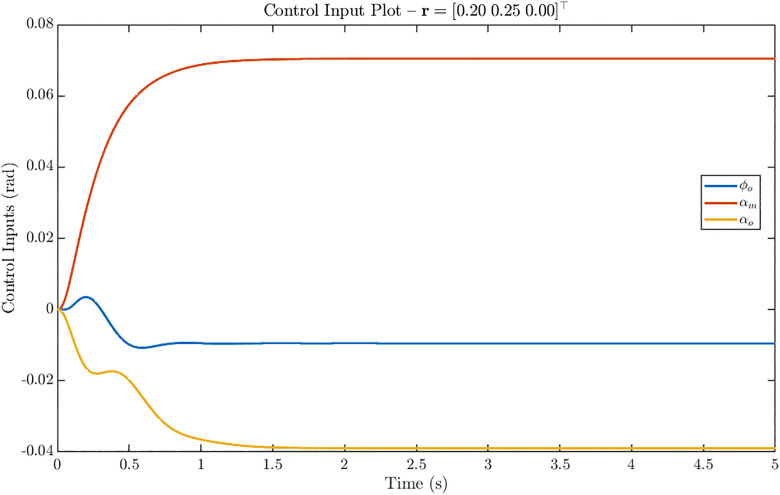
Control input response of ornithopter for reference tracking with r = [0.20 0.25 0]^T^. Control inputs for simultaneous u–w step showing dominant αₘ (≈ 0.07 rad) with compensatory φₒ (≈ −0.01 rad) and αₒ (≈ −0.038 rad), confirming coordinated multi-axis actuation.

Fig 15 shows the control input evolution for the *simultaneous u–w step* reference r = [0.20 0.25 0]^⊤^. Here, all three control channels contribute to meeting the combined tracking demand. The middle channel $\alpha_{m}$ again dominates, settling at about 0.07 rad, while $\varphi_{o}$ and $\alpha_{o}$ provide compensatory actions with steady-state values of approximately −0.01 rad and −0.038 rad, respectively. Both $\varphi_{o}$ and $\alpha_{o}$ exhibit small oscillations during the first 0.5 s before settling, reflecting the coordinated control effort required for multi-axis regulation. These results demonstrate that the $H_{\text{2}}$ controller ensures smooth and well-coordinated actuation across all channels, with efficient distribution of control effort tailored to the commanded motion.

**Fig 16:**
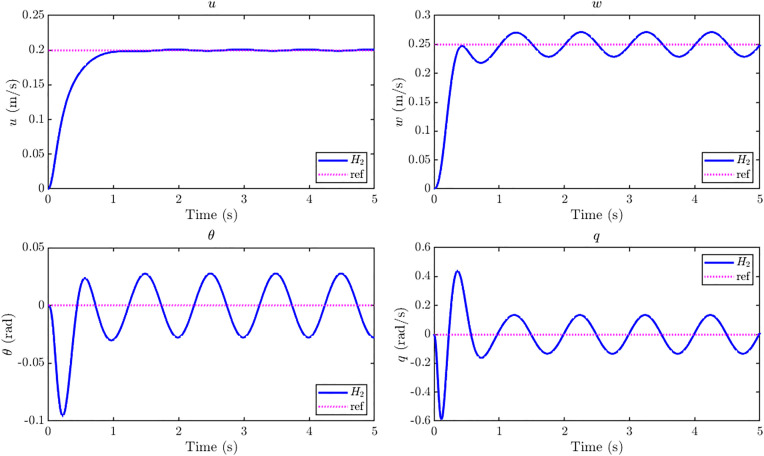
Closed loop state response to sinusoidal gust for reference tracking with r = [0.20 0.25 0]^T^. Closed-loop state responses under sinusoidal gust (amplitude 10, frequency 1 Hz) with H₂ control, showing accurate tracking and well-damped oscillations.

**Fig 17:**
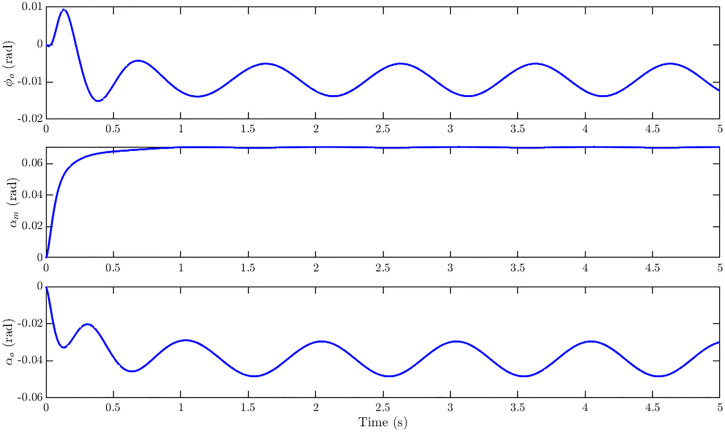
Control input response to sinusoidal gust for reference tracking with r = [0.20 0.25 0]^T^. Control inputs of ornithopter subjected to sinusoidal gust (amplitude 10, frequency 1 Hz) remain smooth and bounded, confirming robust actuation under continuous gust excitation.

Fig 16 illustrates the closed loop longitudinal state responses of the GMS-equipped ornithopter under a vertical sinusoidal gust disturbance of amplitude 10 and frequency 1 Hz, while tracking the step reference r = [0.20 0.25 0]^T^. The $H_{\text{2}}$ controller maintains accurate regulation of the commanded forward velocity *u* and vertical velocity *w*, with only minor oscillatory deviations introduced by the periodic gust. The pitch angle *θ* and pitch rate *q* exhibit well-damped oscillations at the gust frequency, but remain bounded and quickly converge around their equilibrium values. These results confirm that the $H_{\text{2}}$ controller achieves stable reference tracking performance while effectively attenuating the influence of sustained sinusoidal gust inputs. Fig 17 presents the control input trajectories corresponding to the same sinusoidal gust scenario. The smooth and bounded control effort across all channels highlights the efficient distribution of actuation by the $H_{\text{2}}$ controller, ensuring robustness against continuous gust excitation without excessive input demand.

**Fig 18:**
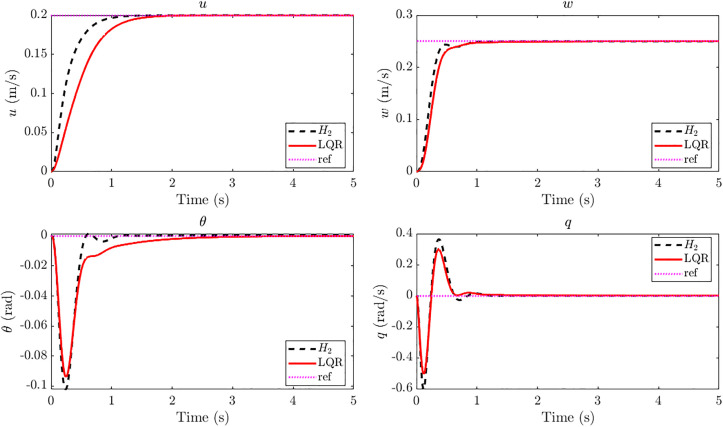
Closed loop states response of *H*_*2*_ controller vs LQR at 20 m/s step gust for reference tracking with r = [0.20 0.25 0]^T^. Closed-loop responses under 20 m/s step gust show that H₂ control improves rise and settling times in u, w, and θ compared to LQR, with zero overshoot and stable convergence within ~1.1 s which is consistent with the experimental findings reported in [15].

Fig 18 shows the GMS-installed ornithopter closed loop responses to a simultaneous step command r = [0.20 0.25 0]^⊤^ under the influence of a step gust disturbance of 20 m/s. Comparison of LQR augmented ornithopter vs $H_{\text{2}}$ augmented ornithopter shows that both controllers achieve effective regulation despite the strong disturbance. Quantitative step metrics show that the $H_{\text{2}}$ design achieves markedly improved transient performance in the primary tracking channels: for forward velocity u, the rise time improves by 38% and the settling time by 33% compared to LQR; for vertical velocity w, the rise time improves by 21% with identical settling; and for pitch angle θ, the settling time is reduced by more than 50%. These gains are achieved with zero overshoot across all three channels. The trade-off is observed in the pitch rate q, where the $H_{\text{2}}$ controller produces a slightly higher peak (0.604 rad/s vs. 0.506 rad/s) and comparable settling of approximately 1 s. Overall, the $H_{\text{2}}$ augmented GMS-equipped ornithopter achieves fast and smooth convergence of the commanded states under severe gust excitation, while maintaining robustness and stability. The closed-loop dynamic state responses in Fig 18 show that all internal states settle within 1.1 s, consistent with the experimental findings reported in [15] where settling time is less than 1.5 s in all simulation scenarios. These results confirm that the designed $H_{\text{2}}$ controller effectively attenuates gust-induced transients through the actuation of the EM feathers, ensuring stable and convergent state trajectories for the GMS-installed ornithopter. The quantitative comparison of LQR vs $H_{\text{2}}$ is presented in Table 3. LQR controller’s design parameters used in this study are provided in the Supporting Information (S8 Table).

**Table 3:** Quantitative comparison of LQR vs *H*_*2*_.

	u		w		θ		q	
	LQR	H_2_	LQR	H_2_	LQR	H_2_	LQR	H_2_
**Rise Time (s)**	0.84	0.52	0.33	0.26	0.20	0.19	0.1	0.1
**Settling Time (s)**	1.45	0.97	0.84	0.84	2.75	1.1	0.92	0.92
**Peak Value**	0.2	0.2	0.25	0.25	−0.093	−0.102	−0.506	−0.601

**Fig 19:**
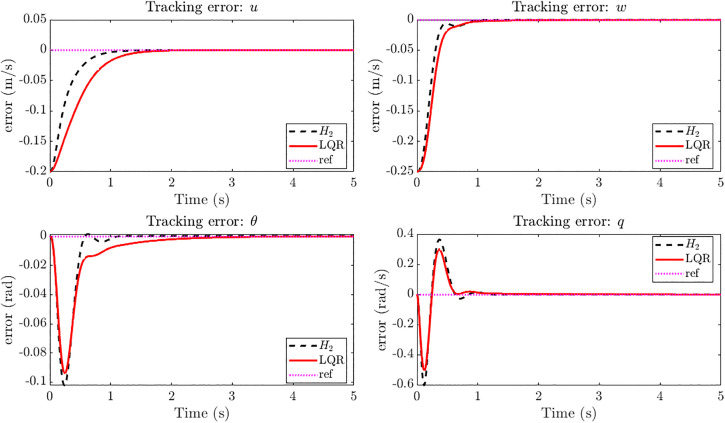
Tracking error of *H*_*2*_ controller vs LQR at 20 m/s step gust for reference tracking with r = [0.20 0.25 0]^T^. Tracking error responses for u, w, θ, and q under LQR and H₂ control, showing faster error decay and improved robustness with H₂.

**Fig 20:**
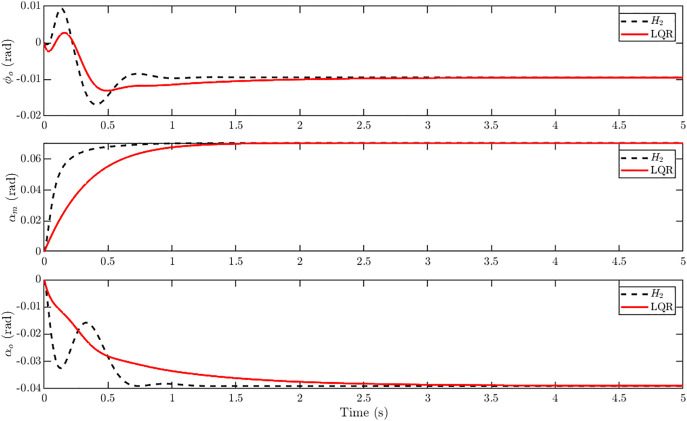
Control effort of *H*_*2*_ controller vs LQR at 20 m/s step gust for reference tracking with r = [0.20 0.25 0]^T^. Comparison of control input responses showing that the H₂ controller settles faster but demands higher initial control effort than the LQR controller.

Fig 19 shows the tracking error responses for u,w,θandq under LQR and $H_{\text{2}}$ control. The $H_{\text{2}}$ controller consistently drives errors to zero more quickly, achieving up to 50% faster settling in u,wandθ. Even in the pitch rate q, where LQR exhibits a slightly smaller peak error, the $H_{\text{2}}$ controller damps the oscillations effectively and ensures overall convergence. These results underline the superior error dynamics of $H_{\text{2}}$ in gust-disturbed tracking, confirming its advantage over LQR in both speed and robustness. The control input profiles in Fig 20 show that the $H_{\text{2}}$ controller achieves faster transients but at the cost of higher initial control effort compared to the LQR controller. All three actuator inputs settle within about 1 s under $H_{\text{2}}$ control, whereas LQR requires approximately 1.3–1.5 s to stabilize. This indicates that the $H_{\text{2}}$ design attains quicker gust rejection and improved responsiveness, albeit with slightly increased control activity.

**Fig 21:**
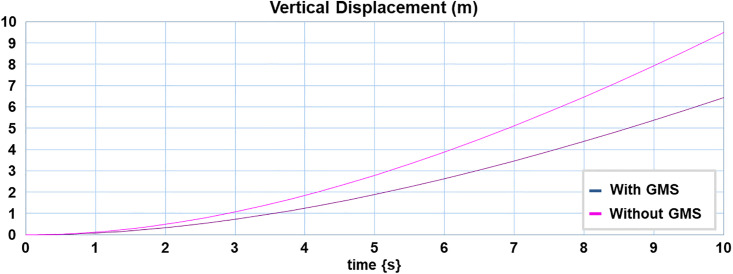
Gust mitigation system (GMS) installed ornithopter vertical displacement at 20 m/s gust. Vertical displacement of ornithopter under 20 m/s step gust showing 32% reduction with GMS and H₂ control actuation, confirming effective gust alleviation by the proposed design.

The displacement in vertical direction of the ornithopter having no GMS and the ornithopter having GMS augmented with designed $H_{\text{2}}$ controller subjected to vertical step gust of 20 m/s is demonstrated in Fig 21. The quantitative values of this comparison are provided in the Supporting Information (S9 Table). The simulation enunciates successful alleviation of gust up to 32% due to actuation of covert feathers along with activation of designed controller as the ornithopter with GMS has a vertical displacement of 6.39 m compared to 9.4 m vertical displacement of ornithopter without GMS. This successfully certifies the efficiency of the proposed design of an ornithopter comprising covert feathers inspired active GMS augmented with $H_{\text{2}}$ controller in tackling gusts.

## 7. Conclusion and future work

This study demonstrated that integrating a kestrel-inspired covert feather–based gust mitigation system (GMS) with an $H_{\text{2}}$ controller enabled stable and resilient flight of a complete ornithopter under turbulent conditions. Using a reduced-order bond-graph model derived from the full flapping-wing dynamics, the controller effectively regulated the longitudinal states and suppressed gust-induced transients by up to 32% compared with the LQR counterpart. Quantitative evaluation showed that all internal states settled within 1.1 s under a 20 m/s gust disturbance, consistent with previously reported experimental results where settling times remained below 1.5 s. These findings confirmed that the integration of bio-inspired aerodynamic mechanisms with optimal $H_{\text{2}}$ control significantly enhanced the robustness and disturbance rejection capability of flapping-wing ornithopters in unsteady flow environments.

The present study is based on a linearized reduced-order model and simplified quasi-steady aerodynamics, which, although effective for control synthesis, do not fully capture the nonlinear unsteady effects encountered in real flapping flight. Future work will address these limitations through higher-fidelity aerodynamic modeling, hardware-in-the-loop and real-time control testing, and experimental validation using a GMS-equipped ornithopter prototype. Additional efforts will also investigate adaptive and learning-based control strategies, as well as energy-consumption and actuator-saturation analyses, to further enhance the practical robustness and efficiency of gust-mitigating ornithopters.

## Supporting information

S1 Table Parameters Values of Bond Graph Model of Main body. These parameter values are vital and used for formulation of the bond graph model of main body of the ornithopter in the Fig 3.
(DOCX)

S2 Table Parameters Values of Bond Graph Model of Motors. These parameter values are vital and used for formulation of the bond graph model of all motors of the ornithopter in the Fig 3.
(DOCX)

S3 Table Parameters Values of Bond Graph Model of Flapping Mechanism. These parameter values are vital and used for formulation of the bond graph model of flapping mechanism of the ornithopter in the Fig 3.
(DOCX)

S4 Table Parameters Values of Bond Graph Model of Rigid Wing. These parameter values are vital and used for formulation of the bond graph model of rigid wing of the ornithopter in the Fig 3.
(DOCX)

S5 Table Parameters Values of Bond Graph Model of Gust Mitigation System. These parameter values are vital and used for formulation of the bond graph model of gust mitigation system of the ornithopter in the Fig 3.
(DOCX)

S6 Table Parameters Values of Bond Graph Model of Gears, Springs and Mechanical Linkages. These parameter values are vital and used for formulation of the bond graph model of gears, springs and mechanical linkages of the ornithopter in the Fig 3.
(DOCX)

S7 Table H_2_ Controller Design Parameters. The H_2_ design parameters used in the research employs mixed-sensitivity weights W1(s) to shape tracking performance and W2(s) to limit control activity, targeting smooth responses with settling times below 1.6 s. These are used in obtaining Figs 8–20.
(DOCX)

S8 Table LQR Controller Design Parameters. The LQR design parameters used in the research provide a balanced trade-off between state regulation and actuator usage while achieving stable control. These are used in obtaining Figs 8–20.
(DOCX)

S9 Table Gust mitigation system (GMS) installed ornithopter vertical displacement at 20 m/s. These are the quantitative values of the vertical displacement of ornithopter with and without GMS subjected to 20 m/s gust that are used to produce the Fig 21.
(DOCX)
