# Supplementary material for: H2 controller design for a kestrel-inspired ornithopter operating in extreme weather
Source: PLoS One. 2026 Feb 12;21(2):e0342245. doi: 10.1371/journal.pone.0342245 (PMC12900442; doi:10.1371/journal.pone.0342245)
Supplement: S1 Table — These parameter values are vital and used for formulation of the bond graph model of main body of the ornithopter in the Fig 3. (DOCX) [file pone.0342245.s001.docx]

**S1 Table. Parameters of the bond graph model of main body**

| **Component** | **Description** | **Values** |
| --- | --- | --- |
| **Main Body** | | |
| Mass of body | Mechanical | 0.15 Kg |
| Mass moment of inertia (J_x_, J_y_, J_z_) | Mechanical | 0.002,0.004,0.003 Kg/m^2^ |
| Gust speed | Mechanical | 25 m/s |
