# Supplementary material for: H2 controller design for a kestrel-inspired ornithopter operating in extreme weather
Source: PLoS One. 2026 Feb 12;21(2):e0342245. doi: 10.1371/journal.pone.0342245 (PMC12900442; doi:10.1371/journal.pone.0342245)
Supplement: S2 Table — These parameter values are vital and used for formulation of the bond graph model of all motors of the ornithopter in the Fig 3. (DOCX) [file pone.0342245.s002.docx]

**S2 Table. Parameters of the bond graph model of motors**

| **Component** | **Description** | **Values** |
| --- | --- | --- |
| **Motors** | | |
| Voltage source | Electrical | 7.2 V |
| Armature resistance of the motors | Electrical | 5.1 Ω |
| Gyrator ratio of motors | Electrical | 0.00813 |
| Damping of motors | Mechanical | 0.00068N-s/m |
| Mass of motors | Mechanical | 0.021Kg |
