# Supplementary material for: H2 controller design for a kestrel-inspired ornithopter operating in extreme weather
Source: PLoS One. 2026 Feb 12;21(2):e0342245. doi: 10.1371/journal.pone.0342245 (PMC12900442; doi:10.1371/journal.pone.0342245)
Supplement: S3 Table — These parameter values are vital and used for formulation of the bond graph model of flapping mechanism of the ornithopter in the Fig 3. (DOCX) [file pone.0342245.s003.docx]

**S3 Table. Parameters of the bond graph model of flapping mechanism**

| **Component** | **Description** | **Values** |
| --- | --- | --- |
| **Flapping Mechanism** | | |
| Mass moment of inertia of crank | Mechanical | 0.009 Kg/m^2^ |
| Transformer ratio of connecting rod | Mechanical | 2 |
| Transformer ratio of linkages | Mechanical | 1 |
| Mass of connecting rod | Mechanical | 0.03 Kg |
| Mass moment of inertia of connecting rod | Mechanical | 0.006 Kg/m^2^ |
