# Supplementary material for: H2 controller design for a kestrel-inspired ornithopter operating in extreme weather
Source: PLoS One. 2026 Feb 12;21(2):e0342245. doi: 10.1371/journal.pone.0342245 (PMC12900442; doi:10.1371/journal.pone.0342245)
Supplement: S5 Table — These parameter values are vital and used for formulation of the bond graph model of gust mitigation system of the ornithopter in the Fig 3. (DOCX) [file pone.0342245.s005.docx]

**S5 Table. Parameters of the bond graph model of GMS**

| **Component** | **Description** | **Values** |
| --- | --- | --- |
| **Flap** | | |
| Mass of flap | Mechanical | 0.018 kg |
| Mass of skeletal structure | Mechanical | 0.098 kg |
| Gust velocity on feather | Mechanical | 25 m/s |
| **Voice Coil Actuator** | | |
| Inductance | Electrical | 0.89 H |
| Stiffness | Mechanical | 0.589 KN/m |
| **Piezoelectric Stack** | | |
| Resistance between amplifier and PZT | Electrical | 5 Ω |
| Mass of Stack | Mechanical | 0.008Kg |
| PZT spring stiffness | Mechanical | 0.024 kN/m |
| PZT equivalent capacitance | Electrical | 1.5x 10 ^-7^ F |
| Coupling Ratio | Electrical | 0.478 |
