# Supplementary material for: H2 controller design for a kestrel-inspired ornithopter operating in extreme weather
Source: PLoS One. 2026 Feb 12;21(2):e0342245. doi: 10.1371/journal.pone.0342245 (PMC12900442; doi:10.1371/journal.pone.0342245)
Supplement: S6 Table — These parameter values are vital and used for formulation of the bond graph model of gears, springs and mechanical linkages of the ornithopter in the Fig 3. (DOCX) [file pone.0342245.s006.docx]

**S6 Table. Parameters of the bond graph model of gear, spring and mechanical linkage**

| **Component** | **Description** | **Values** |
| --- | --- | --- |
| **Gears** | | |
| Ratio of gears | Mechanical | 0.112 |
| **Spring** | | |
| Spring stiffness | Mechanical | 0.03kN/m |
| **Mechanical Linkage** | | |
| Transformer Ratio | Mechanical | 0.2 |
