# Supplementary material for: H2 controller design for a kestrel-inspired ornithopter operating in extreme weather
Source: PLoS One. 2026 Feb 12;21(2):e0342245. doi: 10.1371/journal.pone.0342245 (PMC12900442; doi:10.1371/journal.pone.0342245)
Supplement: S7 Table — The H2 design parameters used in the research employs mixed-sensitivity weights W1(s) to shape tracking performance and W2(s) to limit control activity, targeting smooth responses with settling times below 1.6 s. These are used in obtaining Figs 8–20. (DOCX) [file pone.0342245.s007.docx]

| **Symbol** | **Meaning** | **Value/ Expression** |
| --- | --- | --- |
| W_1_ (s) | Tracking error weight | (s/4+1)/((s/40+1)(s/150+1)) I_3_ |
| W_2_ (s) | Control effort weight | 2(s/10+1)/(s/200+1) I_3_ |
| K_u_ | Control speed factor | 2 |
| T | Reference filter time constant | 0.2 s |
| t_s_ | Desired settling time | <1.6 s |

**S7 Table.** H_2_ Controller Design Parameters
