# Supplementary material for: H2 controller design for a kestrel-inspired ornithopter operating in extreme weather
Source: PLoS One. 2026 Feb 12;21(2):e0342245. doi: 10.1371/journal.pone.0342245 (PMC12900442; doi:10.1371/journal.pone.0342245)
Supplement: S8 Table — The LQR design parameters used in the research provide a balanced trade-off between state regulation and actuator usage while achieving stable control. These are used in obtaining Figs 8–20. (DOCX) [file pone.0342245.s008.docx]

| **Symbol** | **Meaning** | **Value/ Expression** |
| --- | --- | --- |
| Q | State penalty matrix | diag ([0.1 0.1 0.1 0.1]) |
| R | Control penalty matrix | 10 x I_3_ |

**S8 Table.** LQR Controller Design Parameters
