# Supplementary material for: H2 controller design for a kestrel-inspired ornithopter operating in extreme weather
Source: PLoS One. 2026 Feb 12;21(2):e0342245. doi: 10.1371/journal.pone.0342245 (PMC12900442; doi:10.1371/journal.pone.0342245)
Supplement: S9 Table — These are the quantitative values of the vertical displacement of ornithopter with and without GMS subjected to 20 m/s gust that are used to produce the Fig 21. (DOCX) [file pone.0342245.s009.docx]

| **Design** | **Vertical Displacement (m)** |
| --- | --- |
| Ornithopter without GMS | 9.4 |
| Ornithopter with GMS | 6.39 |

**S9 Table.** Gust mitigation system (GMS) installed ornithopter vertical displacement at 20 m/s.
